# Production, Purification, and Characterization of a Cellulase from *Paenibacillus elgii*

**DOI:** 10.3390/polym16142037

**Published:** 2024-07-17

**Authors:** Chien Thang Doan, Thi Ngoc Tran, Thi Phuong Pham, Thi Thanh Thao Tran, Ba Phong Truong, Thi Tinh Nguyen, The Manh Nguyen, Thi Quynh Hoa Bui, Anh Dzung Nguyen, San-Lang Wang

**Affiliations:** 1 Faculty of Natural Science and Technology, Tay Nguyen University, Buon Ma Thuot 630000, Vietnam; dcthang@ttn.edu.vn (C.T.D.); ttngoc@ttn.edu.vn (T.N.T.); ptphuong@ttn.edu.vn (T.P.P.); tttthao@ttn.edu.vn (T.T.T.T.); tbphong@ttn.edu.vn (B.P.T.); nguyentinh@ttn.edu.vn (T.T.N.); ntmanh@ttn.edu.vn (T.M.N.); btqhoa@ttn.edu.vn (T.Q.H.B.); 2 Institute of Biotechnology and Environment, Tay Nguyen University, Buon Ma Thuot 630000, Vietnam; nadzung@ttn.edu.vn; 3 Department of Chemistry, Tamkang University, New Taipei City 25137, Taiwan; 4 Life Science Development Center, Tamkang University, New Taipei City 25137, Taiwan

**Keywords:** cellulase, cellulose, *Paenibacillus elgii*

## Abstract

Cellulases are one of the most essential natural factors for cellulose degradation and, thus, have attracted significant interest for various applications. In this study, a cellulase from *Paenibacillus elgii* TKU051 was produced, purified, and characterized. The ideal fermentation conditions for cellulase productivity were 2% carboxymethyl cellulose (CMC) as the growth substrate, pH = 8, temperature of 31 °C, and 4 days of culturing. Accordingly, a 45 kDa cellulase (PeCel) was successfully purified in a single step using a High Q column with a recovery yield of 35% and purification of 42.2-fold. PeCel has an optimal activity at pH 6 and a temperature of 60 °C. The activity of cellulase was significantly inhibited by Cu^2+^ and enhanced by Mn^2+^. The PeCel-catalyzed products of the CMC hydrolysis were analyzed by high-performance liquid chromatography, which revealed chitobiose and chitotriose as the major products. Finally, the clarity of apple juice was enhanced when treated with PeCel.

## 1. Introduction

Lignocellulosic biomass primarily comprises cellulose, an essential structural element that gives plant tissues stiffness and strength [1]. Cellulose is an unbranched biopolymer comprising glucose units connected by β-1,4-glycosidic linkages [2]. Cellulose chains interact with each other through hydrogen bonds and van der Waals forces, forming highly ordered crystalline structures. Among allomorphic forms, cellulose I is the most commonly found in nature. The crystal structure and hydrogen-bonding arrangement of cellulose I*_α_* and cellulose I*_β_* were determined earlier using atomic-resolution synchrotron and neutron diffraction data [3,4]. More recent spectroscopic studies revealed diverse molecular-level structures of cellulose [5,6]. The transformation of cellulose into fermentable sugars plays a crucial role in providing an abundant carbon supply for fermentation. Despite its abundance, cellulose breakdown remains a significant issue mainly because of its complicated and refractory nature [7]. Striving to achieve a ‘green industry’, it is evident that using enzymes in cellulose conversion is a more ecologically friendly alternative than using chemicals (acid and alkali) [8].

Cellulases, which break down cellulose by splitting the β-1,4-D-glycosidic linkages, are categorized into endoglucanases, exoglucanases, and β-glucosidases [9,10,11]. The synergistic action of these three types of cellulases is crucial to the effective breakdown of cellulose. Endoglucanases shorten cellulose chains by hydrolyzing glycosidic linkages in amorphous cellulose. They also act on cellodextrin, which is formed during the hydrolysis of cellulose. The two main types of exoglucanases include 1,4-β-D-glucan cellobiohydrolase I (CHB I), which acts on the reducing ends of cellulose, and 1,4-β-D-glucan cellobiohydrolase II (CBH II), which targets non-reducing ends, producing cellobiose. Then, the β-glucosidases convert this cellobiose into glucose [12]. Microscopic approaches have been used to understand the mechanisms behind cellulase breakdown in plant cell walls, facilitating the cost-effective transformation of cellulosic biomass [13]. Cellulases are widely used across industries, including paper, textiles, detergents, feed, food, and biofuels [12,14], and account for approximately 20% of the enzyme market [15].

Cellulase is produced by microbes, including fungi and bacteria [2,16], with fungi being the leading cellulase producers, for example, *Aspergillus niger* [17,18] and *Trichoderma reesei* [19]. However, bacteria have recently gained importance as potential cellulase producers [20]. *Paenibacillus*, a former member of the genus *Bacillus* [21], produces various kinds of enzymes [22,23,24,25]. While there is a plethora of published studies on cellulase production by *Paenibacillus* strains [20,21,22,23,24,25,26], reports concerning *P. elgii* are notably scarce. Tran et al. (2024) reported a cellulase-producing strain, *P. elgii* YSY-1.2 [27]. However, the purification and characterization of *P. elgii*’ cellulase is yet to be fully elucidated.

In this research, we investigated the conditions for cellulase biosynthesis by *P. elgii* TKU051 strain and attempted to purify the enzyme. In addition, the expense of enzyme purification is still crucial in bringing products to the market. Accordingly, some studies conduct one-step purification to reduce enzyme costs [28]. In this study, the cellulase was purified in a single step by ion chromatography from the crude enzyme, and its biochemical properties were analyzed. Finally, the potential of the obtained cellulase was assessed in terms of its applications in the clarification of apple juice.

## 2. Materials and Methods

### 2.1. Bacteria Strain

*P. elgii* TKU051 was isolated at Tamkang University (New Taipei, Taiwan) and described in an earlier report [29].

### 2.2. Cellulase Assay

The activity of cellulase form *P. elgii* (PeCel) was assessed in terms of the quantity of reducing sugar liberated from the carboxymethyl cellulose (CMC) hydrolysis. The reaction setup comprised 50 µL of enzyme and 150 µL of 1% CMC prepared in phosphate buffer (pH 6, 100 mM). The mixture was incubated at 60 °C for 30 min. Subsequently, 350 µL of 3,5-dinitrosalicylic acid (DNS) was introduced to stop the reaction, followed by incubation in a water bath at 100 °C for 10 min. The color intensity of the obtained mixture was measured at 540 nm. The negative control was identical to the method described, except the cellulase solution was substituted with a heat-denatured cellulase solution. The cellulase activity was quantified in terms of a unit, which is the enzyme quantity required to generate 1 μmol of reducing sugar as glucose per minute under standard assay conditions.

### 2.3. Screening Fermentation Conditions

The fermentation conditions were screened using the one factor at a time (OFAT) method. The factors included the type of carbon source (sugarcane bagasse powder (SCBP), rice bran powder (RBP), rice husk powder (RHP), corn cob powder (CCP), and carboxymethyl cellulose (CMC)), the amount of CMC (0.25–3%), pH (pH 6–9), temperature (28–40 °C), and incubation time (0–5 days). The basal medium consisted of 0.5% NH_4_NO_3_, 0.1% K_2_HPO_4_, and 0.05% MgSO_4_.

### 2.4. Enzyme Purification

On day 4 of the incubation, 1 L of enzyme solution was harvested from the *P. elgii* TKU051 culture medium by centrifugation (6000 rpm in 20 min). Cold ethanol (−20 °C) was introduced to the enzyme solution in a 3/1 ratio (*v*/*v*) to precipitate the cellulase. The sediment was then solubilized in acetate buffer (50 mM, pH = 5.8) and applied to a High Q column rinsed with the same buffer. Cellulase was eluted by a gradient of sodium chloride (0 to 1 M). The cellulolytic fractions were collected for further experiments. The purity and mass of the obtained enzyme were checked by sodium dodecyl sulfate-polyacrylamide gel electrophoresis (PAGE) [30]. The in-gel cellulolytic activity was examined by zymography using native-PAGE containing 0.05% CMC and 0.1% congo red as the staining reagent.

### 2.5. Enzyme Characterization

The optimum temperature for the activity of PeCel was identified by evaluating its activity across a temperature range of 37 °C to 90 °C. Additionally, the thermal stability of PeCel was determined by incubating it at temperatures from 4 °C to 90 °C for 1 h. To determine the optimal pH, PeCel was evaluated over a pH range of 2.0–10.6 using 50 mM buffers. Simultaneously, the pH stability was studied, and PeCel was incubated at a pH range from 2.0 to 10.6 for 1 h, followed by the estimation of the residual activity of PeCel.

To find out the effect of various chemical additives, PeCel was incubated with each chemical, including NaCl, KCl, ZnCl_2_, FeCl_2_, CaCl_2_, BaCl_2_, MgCl_2_, MnCl_2_, CuCl_2_, E-64, 2-mercaptoethanol (2-ME), ethylenediaminetetraacetic acid (EDTA), 1,10-phenanthroline, and phenylmethanesulfonyl fluoride (PMSF), at a final concentration of 5 mM. Regarding surfactants (cetyltrimethylammonium bromide (CTAB), Triton X-100, sodium dodecyl sulfate (SDS), Tween 20, and Tween 40), a final concentration of 1% was used. This incubation took place at 4 °C for 30 min. The enzyme activity measured with no chemical additions served as the control.

The substrate specificity of PeCel was confirmed using CMC, cellulose powder, chitin, chitosan, xylan, alginate, dextran, gum arabic, pectin, β-1,3-glucan, starch, 2-nitrophenyl-β-D-galactopyranoside, 4-nitrophenyl N-acetyl-β-D-glucosaminide, 4-nitrophenyl-α-D-glucopyranoside, and 4-nitrophenyl-β-D-glucopyranoside. The activity was measured using 1% CMC as the control.

### 2.6. High-Performance Liquid Chromatography and Viscosity Analysis

CMC was used at a 1% concentration to study the hydrolysis mechanism of PeCel. The resulting hydrolysis products were examined at various time intervals, from 0 h to 4 h, using HPLC. The analysis settings included a KS-803 column, water as the mobile phase, and detection by a refractive index detector. The viscosity of the reaction was determined using a rotational viscometer (Brookfield, MA, USA).

### 2.7. Clarification of Apple Juice

The enzyme was added at different concentrations (0–10 U) to the apple juice, and the mixtures were incubated at 50 °C for 2 h. The clarity of the juice was determined by the transmittance (%T) at 660 nm against distilled water. The concentration of reducing sugar was determined using Miller’s method (1959) [31]. The α, α-diphenyl-β-picrylhydrazyl (DPPH) radical scavenging activity was performed according to a previous report [32].

## 3. Results and Discussion

### 3.1. Screening the Cellulase Biosynthesis Conditions

**Figure 1:**
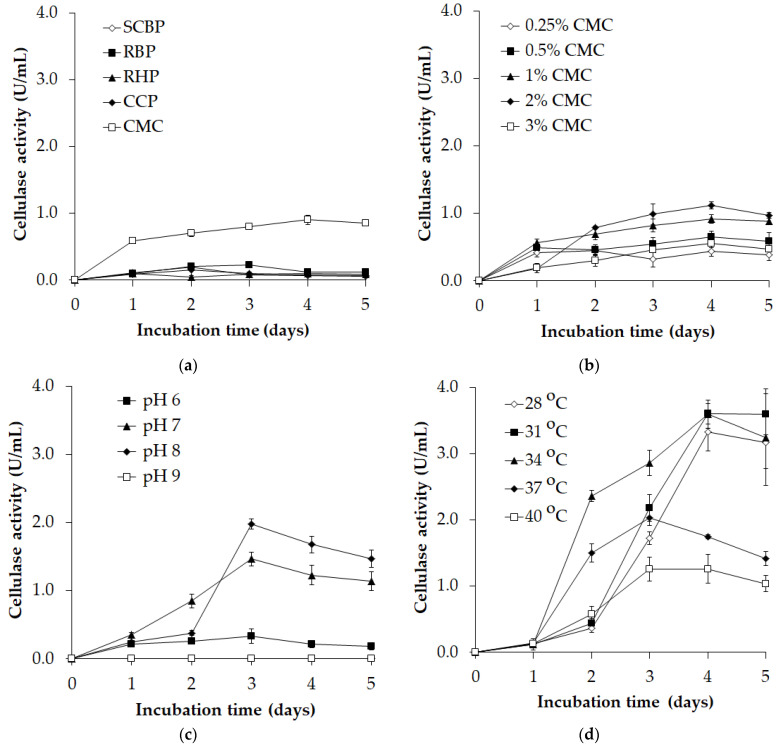
Effect of the carbon source (**a**), CMC ratio (**b**), pH (**c**), and temperature (**d**) on cellulase productivity by *P. elgii* TKU051. Data are the mean of three replications, and the error bars represent the SD (standard deviation).

*P. elgii* TKU051 was cultured on media containing different carbon sources, including SCBP, RBP, RHP, CCP, and CMC to explore the cellulase productivity. This strain showed poor cellulase productivity in the SCBP-, RBP-, RHP-, and CCP-containing media (Figure 1a). The maximum cellulase content was observed in the CMC-containing medium on the fourth day of the incubation (Figure 1a). This result agrees with previous reports that CMC is the best carbon source for cellulase synthesis [33,34,35]. CMC dissolves in water, providing easy access and utilization by bacterial cells, whereas the other substrates remain insoluble. Further assays showed that 2% CMC was most suitable for cellulase biosynthesis, and the maximum cellulase activity was observed on day 4 (Figure 1b). Similarly, the highest cellulase productivity by *P. barcinonesis* was noted when using 2% CMC [20].

The culture parameters of pH and temperature could alter the enzyme productivity [36]. Growth media containing 2% CMC at different pH levels (pH 6, pH 7, pH 8, and pH 9) were prepared. *P. elgii* TKU051 could produce cellulase in the media within the pH range of 6–8, with pH 8 yielding optimum cellulase production (Figure 1c). Previous studies have reported pH 7 as optimum for cellulase production [36,37,38]. Finally, an assessment of the impact of temperature on cellulase productivity revealed the highest cellulase productivity of *P. elgii* TKU051 at 31–34 °C (Figure 1d). This finding is almost identical to that reported by Asha et al. (2012) [20], Boondaeng et al. (2024) [38], and Sohail et al. (2016) [39], who observed that the optimal temperature for cellulase synthesis was 35 °C. At lower temperatures, substrate access by cells is restricted, leading to a decline in enzyme synthesis. At the same time, at higher temperatures, the enzyme activity declines due to thermal denaturation [38]. In this study, the most suitable incubation time for harvesting cellulase was on day 4 (3.601 ± 0.153 U/mL) (Figure 1d).

### 3.2. Enzyme Purification

**Figure 2:**
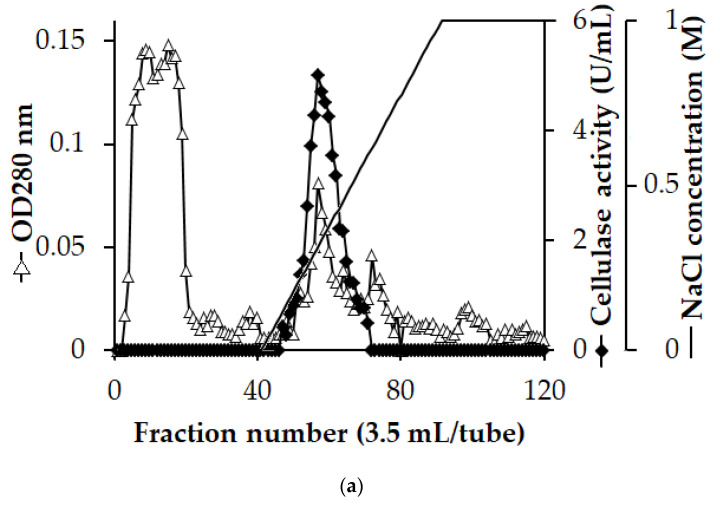

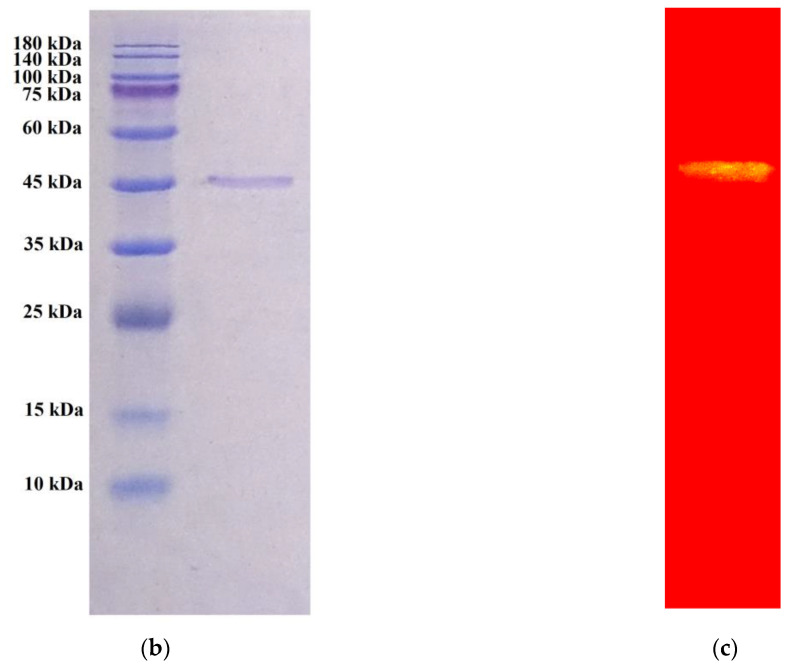
High Q column chromatography profile of the crude cellulase from *Paenibacillus elgii* TKU051. (**a**) Sodium dodecyl sulfate-polyacrylamide gel electrophoresis (**b**) and zymography (**c**) profiles of PeCel.

The crude enzyme was obtained from the culture medium through ethanol precipitation and could be directly subjected to the purification step without the need for salt removal beforehand. The purification process was accomplished in a single step using a High Q column. As shown in Figure 2a, a high amount of protein was washed out before the gradient was applied, and during the elution stage, the protein peak and the cellulase activity peak coincided, suggesting that the purification process may be highly effective. As mentioned in Table 1, the recovery yield of purified cellulase (PeCel) was 35%, with 42.2-fold purification. These recovery yield and fold-purification values were better than the values reported by Dar et al. (2019) [40], Elsababty et al. (2022) [41], and Nisar et al. (2022) [10]. The specific activity of the obtained cellulase was 358.7 U/mg, surpassing that of *P. barcinonensis* (16.88 U/mg) [20] and comparable to that of *B. licheniformis* Z9 cellulase (356.5 U/mg) [41].

The activity fraction from the chromatography step revealed a unique protein band of 45 kDa on SDS-polyacrylamide gel (Figure 2b). Zymography analysis reveals a distinct band on the congo red theme, confirming the cellulolytic activity of the purified cellulase (Figure 2c). Therefore, a cellulase of 45 kDa molecular weight was successfully purified in a single step using a High Q column. As illustrated in Table 2, the MW of *P. elgii* TKU051 cellulase differs from those produced by other *Paenibacillus* and *Bacillus* strains. Only a cellulase of *Bacillus tequilensis* G9 (43 kDa) [40] closely resembles that obtained in this study. This could be a novel cellulase produced by the *Paenibacillus* genus.

### 3.3. Enzyme Characterization

**Figure 3:**
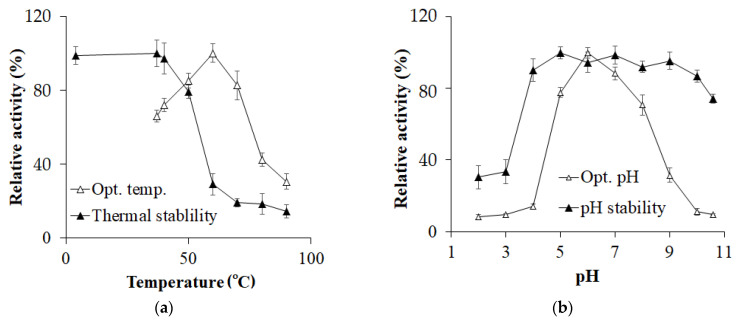
The impact of temperature (**a**) and pH (**b**) on the activity of *Paenibacillus elgii* TKU051 cellulase (PeCel).

The PeCel obtained in this study exhibited optimal activity at 60 °C and remained stable up to 40 °C (Figure 3a). After heating the purified enzyme at 50 °C for 1 h, approximately 80% of its activity was retained. The optimal temperature of 60 °C is in close agreement with that for cellulases produced by *P. polymyxa* GS01 [41], *P. barcinonensis* [20], *P. campinasensis* BL11 [46], and *B. licheniformis* ATCC 14580 [48]. Figure 3b shows that PeCel exhibits optimal activity at pH 6. The optimal pH range of cellulase from *Paenibacillus* seems to be in the range of 5.0–7.0 (Table 2). The activity of PeCel stabilized over the pH range of 4–10.

The substrate specificity of PeCel was explored by utilizing different kinds of polysaccharides as substrate, including CMC, cellulose powder, chitin, chitosan, xylan, alginate, dextran, gum arabic, pectin, β-1,3-glucan, and starch. The enzyme activity was the highest for CMC (100.00 ± 4.65%), followed by cellulose powder (29.47 ± 0.58%). This result is in agreement with those reported by Kim et al. (2023) [8] and Ko et al. (2010) [46]. PeCel could also use other polysaccharide substrates (chitin, chitosan, xylan, alginate, dextran, gum arabic, pectin, β-1,3-glucan, and starch), albeit to a lesser extent (Table 3). In addition, four types of chromogenic substrates (2-nitrophenyl-β-D-galactopyranoside, 4-nitrophenyl N-acetyl-β-D-glucosaminide, 4-nitrophenyl-α-D-glucopyranoside, and 4-nitrophenyl-β-D-glucopyranoside) were also used to assess the substrate specificity of PeCel. PeCel did not catalyze the cleavage activity on all four chromogenic substrates (Table 3), suggesting that it may lack β-galactosidase, β-glucosaminidase, α-glucosidase, and β-glucosidase activities. In fact, Lee et al. (2008) also indicated that the endo-type glucanase from *B. amyloliquefaciens* DL-3 could not hydrolyze 4-nitrophenyl-β-D-glucopyranoside [9].

Table 4 presents the effect of various chemicals on the activity of PeCel. Considering the effect of metal ions, the enzyme activity was significantly enhanced and reduced in the presence of Mn^2+^ (140.00 ± 0.27%) and Cu^2+^ (35.88 ± 2.64%), respectively. The impact of metal ions on cellulase activity may appear to differ among various bacterial strains. Indeed, in stark contrast, Cu^2+^ was reported to act as an activator while Mn^2+^ served as an inhibitor of cellulases from *P. peoriae* MK1 [4], *P. xylanilyticus* KJ-03 [38], and *B. licheniformis* Z9 [41]. Enzyme inhibitors (E-64, EDTA, PMSF, and 1,10-phenanthroline) and a reducing agent (2-ME) mildly affected the activity of PeCel with the residual activity in the range of 82.16–88.89%. According to recent studies, EDTA could inhibit more than 50% of cellulase activity from *B. tequilensis* SB125 [36] and *B. subtilis* [49]. Among the examined surfactants, the anionic one (SDS) exerted a significant inhibition effect on the activity of PeCel, resulting in a residual activity of 15.39%. In contrast, the effect of cationic surfactant (cetyltrimethylammonium bromide or CTAB) was relatively mild (86.13 ± 3.14%), while the non-ionic surfactants (Triton X-100 and Tween 20) slightly enhanced the activity (117.98 ± 5.19% and 104.34 ± 3.33, respectively). However, Tween 40 (a non-ionic surfactant) decreased the activity of PeCel slightly (90.38 ± 5.20%). Likewise, the stimulatory effect of Triton X-100 and the inhibition effect of SDS on the activity of cellulases have been reported by Malik and Javed (2024) [36].

**Figure 4:**
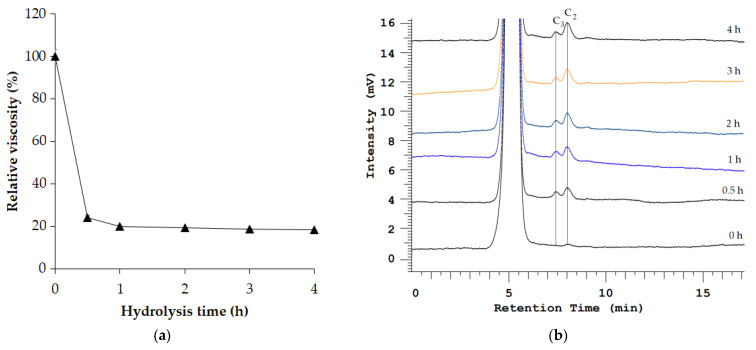
Viscosity profile (**a**) and high-performance liquid chromatography profile (**b**) of carboxymethyl cellulose (CMC) hydrolysates catalyzed by *Paenibacillus elgii* TKU051 cellulase (PeCel). C_2_, cellobiose; C_3_, cellotriose.

The mode of action of PeCel was investigated by incubating this enzyme with the substrate CMC for periods ranging from 0 to 4 h. The viscosity profile of the hydrolysate is presented in Figure 4a. After 0.5 h of hydrolysis, PeCel could decrease the CMC viscosity by over 75%, suggesting its endo-acting function [50]. The HPLC profile of the hydrolysate is shown in Figure 4b. Initially, at 0 h, no product peaks were observed; however, from 0.5 h onward, two peaks emerged (8.05 min and 7.45 min), representing those of cellobiose and cellotriose (respectively). These results indicate that PeCel hydrolyzes CMC to primarily produce two products: cellotriose (C_3_) and predominantly cellobiose (C_2_). In contrast to exoglucanases, cellulose hydrolysis by endoglucanases results in a mixture of products [51]. Wu et al. (2018) reported that a GH 5 family endoglucanase from *B. subtilis* BS-5 could hydrolyze cellulose to produce cellobiose and cellotriose [50]. Likewise, another GH 5 family endoglucanase from *Cytophaga hutchinsonii* could also hydrolyze cellulose to produce cellobiose and cellotriose as the main products [52]. This result suggests that PeCel may be an endoglucanase.

### 3.4. Clarification of Apple Juice

The viscosity and turbidity of a juice are related to the presence of cellulose [53], which can be clarified by cellulase [14,54]. After cellulase treatment, the clarification values for apple juice (T%) improved and were directly related to the enzyme concentration. In other words, the clarity of apple juice increased by 34.54 ± 0.62%, 62.52 ± 1.54%, 68.19 ± 1.85%, and 71.33 ± 1.92% upon treatment with 0 U, 1 U, 5 U, and 10 U of PeCel, respectively. The amount of reducing sugar in apple juices treated with different enzyme concentrations (0 U, 1 U, 5 U, and 10 U) increased by 0.82 ± 0.08 mM, 1.02 ± 0.09 mM, 1.31 ± 0.07 mM, and 1.51 ± 0.10 mM, respectively.

Apples are abundant in antioxidants [55], and their fruit possesses significant free radical scavenging ability [56]. Thus, this study also investigated the effect of PeCel-mediated enzymatic clarification on the DPPH scavenging ability of apple juice. Table 5 shows that the difference between the DPPH radical scavenging activity values of apple juice treated with different enzyme concentrations was non-significant. Studies have reported that enzymatic treatment may have a detrimental influence on antioxidant activity [54,57]. The antioxidant ability of a fruit is linked to the ascorbic acid, phenolic, and flavonoid contents, and the clarifying process may cause the deposition of scavengers such as phenolics. In contrast, Chen et al. (2023) found that treatment by pectinase enhances the antioxidant ability of guava juices [58]. Pectinases degrade the cell wall, thereby releasing polyphenols and flavonoids, improving the antioxidant content and activity in the juice.

## 4. Conclusions

*P. elgii* TKU051 could synthesize the cellulase PeCel with the highest productivity of 3.601 U/mL in a medium containing 2% CMC as the sole carbon source. This cellulase, isolated and purified from the culture medium in a single step, exhibited a molecular weight of 45 kDa and was biochemically characterized. PeCel exhibited optimal activity at pH 6.0 and 60 °C and was considered an endoglucanase, as it efficiently catalyzed the conversion of CMC into cellobiose and cellotriose as the main products. Moreover, PeCel also significantly improved the clarification of apple juice, indicating its high potential for usage in the fruit juice industry.

## Figures and Tables

**Table 1:** A summary of the purification of cellulase produced by *Paenibacillus elgii* TKU051.

Step	Total Protein (mg)	Total Activity (U)	Specific Activity (U/mg)	Recovery (%)	Purification (fold)
Culture supernatant	519.5	4419.3	8.5	100	1.0
EtOH precipitation	262.6	3652.1	13.9	83	1.6
High Q column	4.3	1554.5	358.7	35	42.2

**Table 2:** Properties of cellulases from different *Paenibacillus* and *Bacillus* strains.

Cellulase-Producing Strain	MW (kDa)	Opt. pH	Opt. Temp. (°C)	Metal Ion		Ref.
				Inhibitor	Activator	
*Paenibacillus elgii* TKU051	45	6.0	60	Cu^2+^	Mn^2+^	This study
*Paenibacillus peoriae* MK1	65	5.0	40	Mn^2+^	Cu^2+^, Ba^2+^, Mg^2+^, and Fe^2+^	[8]
*Paenibacillus terrae* ME27-1		5.5	50			[42]
*Paenibacillus polymyxa* GS01	33	7.0	50			[43]
	60	6.0	60			
*Paenibacillus xylanilyticus* KJ-03	64	5.0	40	Fe^3+^, Mn^2+^, Zn^2+^, and Hg^2+^	Ca^2+^ and Cu^2+^	[44]
*Paenibacillus* sp. IHB B 3084	63.5		40			[45]
*Paenibacillus barcinonensis*	58.6	6.0	65	Hg^2+^	Mg^2+^, Fe^2+^, Mn^2+^, and Zn^2+^	[20]
*Paenibacillus campinasensis* BL11	38	7.0	60	Hg^2+^, Cu^2+^, and Zn^2+^	Mn^2+^ and Co^2+^	[46]
*Paenibacillus* sp.	67	7.0	40			[33]
*Bacillus licheniformis* Z9	54.4	7.4	30	Mg^2+^ and Na^+^	Fe^3+^, Cu^2+^, and Ca^2+^	[41]
*Bacillus amyloliquefaciens* FW2	55			Fe^2+^ and Zn^2+^	Mg^2+^ and Ca^2+^	[47]
*B. licheniformis* ATCC 14580	61.7	7.0	60			[48]
*B. tequilensis* G9	43	5.0	40	Pb^2+^	Zn^2+^, Ca^2+^, and Co^2+^	[40]

**Table 3:** Substrate specificity of the obtained cellulase (PeCel).

Substrate	Relative Activity
CMC *	100.00 ± 4.65
Cellulose powder	29.47 ± 0.58
Chitin	12.44 ± 0.50
Chitosan	23.96 ± 3.06
Xylan	20.26 ± 0.75
Alginate	19.44 ± 1.19
Dextran	19.82 ± 1.05
Gum arabic	17.04 ± 0.48
Pectin	17.83 ± 1.60
β-1,3-Glucan	11.51 ± 1.02
Starch	5.69 ± 0.56
2-Nitrophenyl β-D-galactopyranoside	N.D.
4-Nitrophenyl N-acetyl-β-D-glucosaminide	N.D.
4-Nitrophenyl-β-D-glucopyranoside	N.D.
4-Nitrophenyl-α-D-glucopyranoside	N.D.

*, control; N.D., not detected.

**Table 4:** Effect of various chemicals on the obtained cellulase (PeCel).

Chemical	Relative Activity
Control	100 ± 3.06
Na^+^	97.57 ± 8.22
K^+^	96.81 ± 2.45
Zn^2+^	91.82 ± 6.87
Fe^2+^	90.23 ± 2.05
Ca^2+^	97.44 ± 5.18
Ba^2+^	101.46 ± 6.25
Mg^2+^	101.18 ± 3.39
Mn^2+^	140.00 ± 0.27
Cu^2+^	35.88 ± 2.64
E64	82.16 ± 5.30
2-ME	88.89 ± 7.04
EDTA	86.36 ± 2.31
1,10-Phenanthroline	82.68 ± 5.22
PMSF	88.56 ± 8.24
SDS	15.39 ±5.50
CTAB	86.13 ± 3.14
Triton X-100	117.98 ± 5.19
Tween 20	104.34 ± 3.33
Tween 40	90.38 ± 5.20

**Table 5:** Effect of PeCel on apple juice properties.

	Enzyme Concentration			
	0 U	1 U	5 U	10 U
Clarification (T%)	34.54 ± 0.62	62.52 ± 1.54	68.19 ± 1.85	71.33 ± 1.92
Reducing sugar (mM)	0.82 ± 0.08	1.02 ± 0.09	1.31 ± 0.07	1.51 ± 0.10
DPPH radical scavenging activity (%)	72.78 ± 1.83	71.57 ± 1.45	70.92 ± 1.30	70.02 ± 1.86

## Data Availability

Data are contained within the article.
